# The Caloric Test Is More Consistent With the Presence of Endolymphatic Hydrops Than the Vestibular-Evoked Myogenic Potential Test in Meniere's Disease

**DOI:** 10.7759/cureus.51384

**Published:** 2023-12-31

**Authors:** Toshitake Tanaka, Munetaka Ushio, Hitoshi Terada, Taro Takanami, Seikei Kan, Hiroaki Masuda, Kotaro Ochi, Hitomi Ikeda, Ryosuke Yoshino, Yasushi Ohta

**Affiliations:** 1 Otolaryngology, Toho University Sakura Medical Center, Sakura, JPN; 2 Radiology, Toho University Sakura Medical Center, Sakura, JPN

**Keywords:** vestibular evoked myogenic potential, caloric test, endolymphatic hydrops, meniere’s disease, magnetic resonance imaging

## Abstract

Objective

This study aimed to investigate the correlation between enhanced inner ear magnetic resonance imaging (MRI) findings and vestibular and cochlear function test results in patients with definite Meniere’s disease and confirmed endolymphatic hydrops.

Methods

Among 70 consecutive patients diagnosed with definite Meniere’s disease, 49 underwent contrast-enhanced 3-T inner ear MRI. The patients also underwent pure-tone audiometry, glycerol, caloric, and vestibular-evoked myogenic potential (VEMP) tests. Correlations between the pure-tone audiometry, glycerol test, caloric test, VEMP test, and MRI findings were evaluated using the chi-square test or Fisher’s exact test, Student’s t-test, one-way ANOVA, and Bonferroni’s post-hoc test.

Results

Contrast-enhanced inner ear MRI revealed that 33 of 49 patients (67.3%) had endolymphatic hydrops. Among them, 19 patients had bilateral endolymphatic hydrops, and 14 had unilateral hydrops. The mean hearing threshold was higher in patients with endolymphatic hydrops than those without (p< 0.001). The proportion of patients with positive glycerol test results was higher among those with endolymphatic hydrops than in those without (p= 0.01). The rate of abnormal caloric response in patients with and without endolymphatic hydrops was not significantly different (p= 0.09). Furthermore, the rate of abnormal VEMP response in patients with and without endolymphatic hydrops was not significantly different (p= 0.70). On the affected side, in the caloric test, the ratio of the presence of vestibular and cochlear hydrops was similar (p= 1.00). On the affected side, in the VEMP test, the ratio of the presence of vestibular and cochlear hydrops was also similar (p= 0.80). The consistency of the caloric test in detecting cochlear hydrops was higher than that of the VEMP test (p= 0.04). The consistency of the caloric test in detecting vestibular hydrops tended to be higher (but not significantly) than that of the VEMP test (p= 0.11).

Conclusion

The cochlea and vestibule on the clinically affected side were more likely to have endolymphatic hydrops revealed by contrast-enhanced 3-T inner ear MRI than on the unaffected side. The sum of the three low frequencies (125, 250, and 500 Hz) of the pure-tone audiometry was higher in patients with endolymphatic hydrops than in those without endolymphatic hydrops. The caloric test was more consistent in detecting endolymphatic hydrops, especially cochlear hydrops, than the VEMP test in patients with definite Meniere's disease. The results of this study may contribute to the future diagnosis of Meniere's disease and improve the understanding of endolymphatic hydrops.

## Introduction

Meniere's disease is associated with endolymphatic hydrops [1] and causes recurrent spontaneous episodic vertigo and fluctuating hearing impairment. Cochlear and vestibular symptoms are mild in the early stages of the disease, whereas recurrent attacks may lead to severe hearing impairment and balance disorders. In the past, Meniere's disease was diagnosed based on patient history, including recurrent vertigo attacks accompanied by cochlear symptoms, such as hearing impairment and tinnitus, without morphological evaluation [2]. Recently, contrast-enhanced 3-T inner ear magnetic resonance imaging (MRI) has allowed the distinction between signals from the endolymphatic space and those from the perilymphatic space of the inner ear [3]. The contrast agent administered intravenously reaches the perilymph but not the endolymph, allowing the endolymph to be identified as a low-intensity area [3]. Therefore, the presence of endolymphatic hydrops can be confirmed as an enlarged low-intensity area following inner ear MRI.

Many reports show the correlation between enhanced inner ear MRI findings and cochlear function in patients with Meniere’s disease. However, the correlations between MRI findings and vestibular function have rarely been reported [4,5]. Meniere's disease impairs the cochlear and vestibular organs. Still, the correlations between inner ear MRI findings, which can detect endolymphatic hydrops, and vestibular function test results need to be investigated.

This study investigated the correlation between enhanced inner ear MRI findings and vestibular and cochlear function test results in patients with definite Meniere’s disease and confirmed endolymphatic hydrops.

## Materials and methods

Ethical statement

This retrospective, non-randomized, single-group study conducted at the Toho University Sakura Medical Center was approved by the Institutional Ethics Committee (approval number: S19029). Informed consent was obtained from all patients. This study conformed to the Code of Ethics of the World Medical Association (Declaration of Helsinki).

Patients

**Figure 1:**
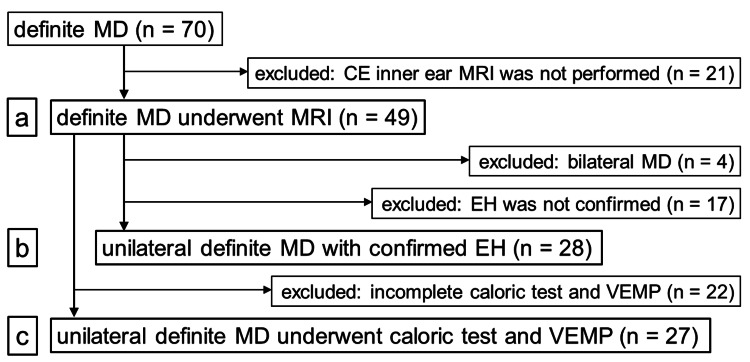
Selection process for the patients included in this study MD, Meniere’s disease; CE, contrast-enhanced; MRI, magnetic resonance imaging; EH, endolymphatic hydrops; VEMP, vestibular-evoked myogenic potential.

The patients comprised 70 consecutive patients (45 women and 25 men) diagnosed with definite Meniere’s disease between June 2016 and October 2019. Among them, 49 (31 women and 18 men) underwent contrast-enhanced 3-T inner ear MRI (Figure 1) [3,6].

The mean ± standard deviation (SD) of their age was 55.7 ± 15.0 years (range, 17-76 years) at the time of examination. The patients were diagnosed based on the 1995 criteria of the American Academy of Otolaryngology-Head and Neck Surgery (AAO-HNS) [2]. Among the 49 patients, 45 were clinically diagnosed with unilateral definite Meniere’s disease, and four had bilateral Meniere’s disease.

Methods

Contrast-enhanced 3-T inner ear MRI was performed (MAGNETROM® Skyra 3T, Siemens, Erlangen, Germany). The MRI methods have been described previously [3,6]. The patients underwent magnetic resonance scanning four hours after a single-dose intravenous injection of meglumine gadoterate (Magnescope, Guerbet Japan KK, Tokyo, Japan). The ratio of the area of the endolymphatic space in the lymphatic space of the cochlea and that in the vestibule was defined as %EL = (number of negative pixels for the endolymph)/(total number of pixels for the endolymph and perilymph) [6]. It was determined that endolymphatic hydrops was present when the %EL was greater than 33.3%. Within a week before the MRI, the patients underwent pure-tone audiometry, caloric, vestibular-evoked myogenic potential (VEMP), and glycerol tests.

Pure-tone audiometry was performed using an audiometer (Audiometer AA-78, RION Co., Ltd., Tokyo, Japan). The sum of the three low frequencies (125, 250, and 500 Hz) and the arithmetic mean of five frequencies (250, 500, 1000, 2000, and 4000 Hz) on pure-tone audiometry were employed to evaluate the hearing level.

A bithermal caloric test was performed using 46ºC warm and 26ºC cold air for 60 seconds. Percent canal paresis (CP) was calculated with the following formula using the maximum slow-phase eye velocity of induced nystagmus, based on the Jongkees' formula:

\begin{equation*} \text{Percent canal paresis (CP)} = \frac{100 \left| (RC + RW) - (LC + LW) \right|}{(RC + RW + LC + LW)} \end{equation*}

Where RW represents the maximum slow-phase eye velocity of induced nystagmus when the right ear was stimulated with warm air; RC represents the right ear with cold air; LW represents the left ear with warm air; and LC represents the left ear with cold air.

The tests were performed using an electronystagmograph (Nystagmograph NY-50, RION Co., Ltd., Tokyo, Japan) in the supine position (head up by 30°) in a completely dark room. The caloric response was considered abnormal when the CP was 20% or more.

VEMP tests [7] were performed in response to short tone bursts (500 Hz at 95 dBnHL; rise/fall time, 1 ms; plateau time, 2 ms; burst VEMPs) using Neuropack Σ (Nihon Kohden, Tokyo, Japan). Surface electromyographic (EMG) activity was recorded from symmetrical sites over the upper half of each sternocleidomastoid muscle (SCM), with reference electrodes on the lateral end of the upper sternum. The ground electrode was placed at the nasion. During the recording of the burst VEMPs, the patients were instructed to raise their heads to activate the SCM, and the EMG activity of the bilateral SCM was monitored. In all enrolled patients, normal SCM activity was observed following recordings on both sides, indicating normal accessory nerve function. The VEMP responses were regarded as abnormal when the responses on the affected side were absent or decreased compared to those on the unaffected side. For this comparison, the percent VEMP asymmetry [7] was calculated using the following formula with an amplitude of p13 - n23 on the affected side (Aa) and the unaffected side (Au):

\begin{equation*} \text{Percent VEMP asymmetry} = \frac{100 \left| \displaystyle (Au - Aa) \right|}{Au + Aa} \end{equation*}

The upper limit of the percent VEMP asymmetry was set at 34.1, according to a previous report [7].

For the glycerol test, pure-tone audiometry was performed before glycerin administration. One milliliter of glycerin per kilogram of body weight was taken, and pure-tone audiometry was performed one, two, and three hours later. The glycerol test [8] was considered positive when an improvement in the pure-tone hearing of 10 dB at an average hearing level of 125, 250, and 500 Hz or more was observed three hours after the administration of glycerin.

Correlations among the disease duration, pure-tone audiometry, glycerol test, caloric test, VEMP test, and MRI findings were evaluated (Figure 1A).

To evaluate the correlation between the presence of endolymphatic hydrops and the clinically affected side, we limited to 28 patients with unilateral Meniere's disease and confirmed endolymphatic hydrops on at least one side to compare the clinically affected and unaffected sides (Figure 1B).

To evaluate the consistency of detecting endolymphatic hydrops with the caloric and VEMP tests, 22 of the 49 patients were excluded because of an incomplete caloric or VEMP test (Figure 1C). The results of the caloric and VEMP tests were considered consistent with the presence of endolymphatic hydrops when the endolymphatic hydrops was present on at least the affected side of the caloric and VEMP tests or when the tests were normal, and there was no endolymphatic hydrops on either side.

Statistical analyses were performed using SPSS 22.0 statistical software (IBM Corp., Armonk, NY). The proportion of patients was compared using the chi-square test or Fisher’s exact test. Mean values were compared using Student’s t-test. One-way ANOVA was used for multigroup comparisons, and Bonferroni’s post-hoc test was also conducted. The data are presented using descriptive statistics: means ± SD. Differences of p < 0.05 were considered significant.

## Results

Contrast-enhanced inner ear MRI revealed that 33 of the 49 patients (67.3%) had endolymphatic hydrops. Among them, 19 had bilateral endolymphatic hydrops, and 14 had unilateral hydrops, i.e., endolymphatic hydrops was observed in 52 of the 98 ears (53.1%).

Duration of the disease and MRI findings

The mean disease duration was 144.1 ± 146.3 months for patients with unilateral endolymphatic hydrops (n = 14), 78.3 ± 59.2 months for patients with bilateral endolymphatic hydrops (n = 12), and 48.3 ± 58.0 months for patients without endolymphatic hydrops (n = 23). The mean disease duration differed significantly among the three groups (p = 0.04). The mean disease duration in patients with unilateral endolymphatic hydrops was longer than that in those without endolymphatic hydrops (p = 0.005). However, the difference in the mean disease duration between patients with unilateral endolymphatic hydrops and those with bilateral endolymphatic hydrops (p = 0.16) and the difference between patients with bilateral endolymphatic hydrops and those without endolymphatic hydrops (p = 0.97) were both not significant.

Pure-tone audiometry and MRI findings

The sum of the three low frequencies (125, 250, and 500 Hz) of the pure-tone audiometry was higher in patients with endolymphatic hydrops (128.1 ± 65.3 dB) than in those without endolymphatic hydrops (66.7 ± 34.5 dB) (p < 0.001). The mean hearing threshold was higher in patients with endolymphatic hydrops (42.1 ± 23.0 dB) than in those without endolymphatic hydrops (17.6 ± 13.8 dB) (p < 0.001).

Glycerol test and MRI findings

Glycerol tests were performed in 17 patients. Positive glycerol test results were observed in five of eight cases (62.5%) of patients with endolymphatic hydrops; however, no positive glycerol test result was observed in the nine patients without hydrops (0%). The proportion of patients with positive glycerol test results was higher among those with endolymphatic hydrops than among those without (p = 0.01).

Caloric test and MRI findings

Caloric tests were performed in 37 patients (74 ears). The mean maximum slow-phase eye velocity did not differ between the 33 ears with endolymphatic hydrops (26.08 ± 15.16) and 41 ears without hydrops (21.50 ± 10.57) (p = 0.13). The rate of the abnormal caloric response in the patients with endolymphatic hydrops (15/22, 68.2%) was higher than that in those without endolymphatic hydrops (6/15, 40.0%); however, the difference was not significant (p = 0.09).

VEMP test and MRI findings

Cervical VEMP tests were performed in 37 patients (74 ears). The mean amplitudes of VEMPs did not differ between the 33 ears with endolymphatic hydrops (278.02 ± 125.44) and 41 ears without hydrops (240.12 ± 121.64) (p = 0.29). The rate of the abnormal VEMP response in the patients with endolymphatic hydrops (3/22, 13.6%) was not different from that in those without endolymphatic hydrops (3/15, 20.0%) (p = 0.70).

Correlation of the presence of endolymphatic hydrops with the clinically affected side

**Figure 2:**
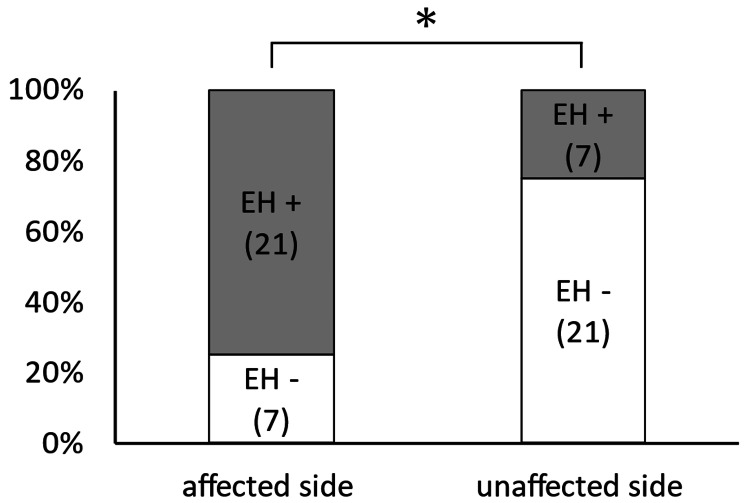
Correlation of the presence of cochlear endolymphatic hydrops with the clinically affected side The cochlea on the clinically affected side was more likely to have endolymphatic hydrops than the cochlea on the unaffected side (p = 0.02). EH, endolymphatic hydrops; *, p < 0.05.

The cochlea on the clinically affected side (21/28, 75%) was more likely to have endolymphatic hydrops than the cochlea on the unaffected side (7/28, 25%) (p = 0.02) (Figure 2).

**Figure 3:**
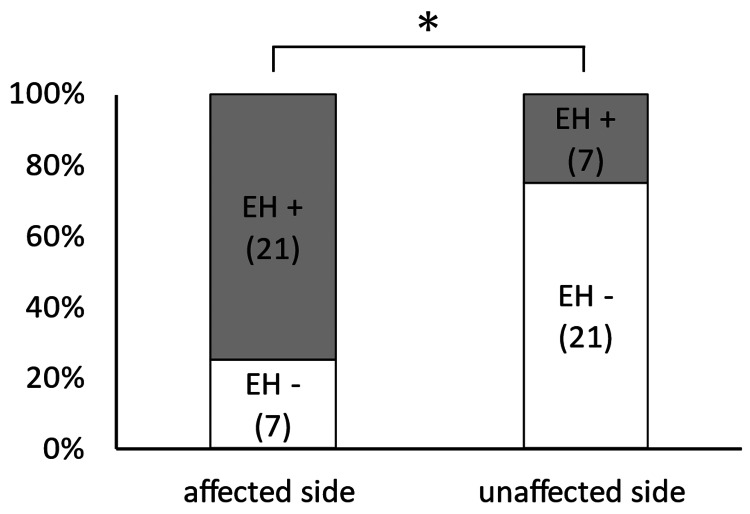
Correlation of the presence of vestibular endolymphatic hydrops with the clinically affected side The vestibule on the clinically affected side was more likely to have endolymphatic hydrops than the vestibule on the unaffected side (p = 0.02). EH, endolymphatic hydrops; *, p < 0.05.

The vestibule on the clinically affected side (21/28, 75%) was also more likely to have endolymphatic hydrops than the vestibule on the unaffected side (7/28, 25%) (p = 0.02) (Figure 3).

Consistency in the detection of endolymphatic hydrops using the caloric and VEMP tests

**Figure 4:**
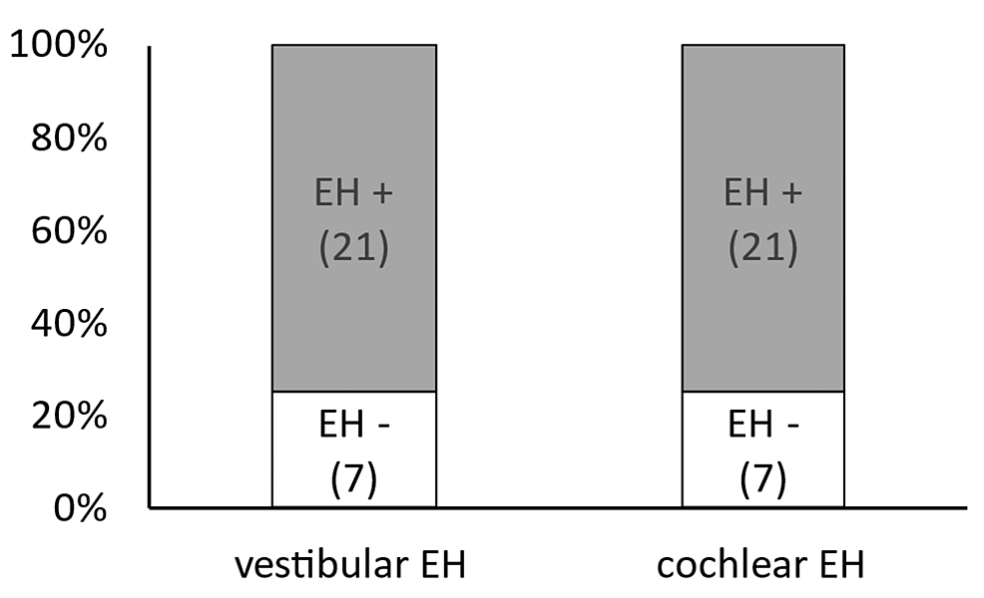
Consistency in the detection of endolymphatic hydrops with the caloric test On the affected side in the caloric test, the ratios of the presence of vestibular and cochlear hydrops were similar (p = 1.00). EH, endolymphatic hydrops.

On the affected side in the caloric test, the ratios of the presence of vestibular (17/27, 63.0%) and cochlear (17/27, 63.0%) hydrops were similar (p = 1.00) (Figure 4).

**Figure 5:**
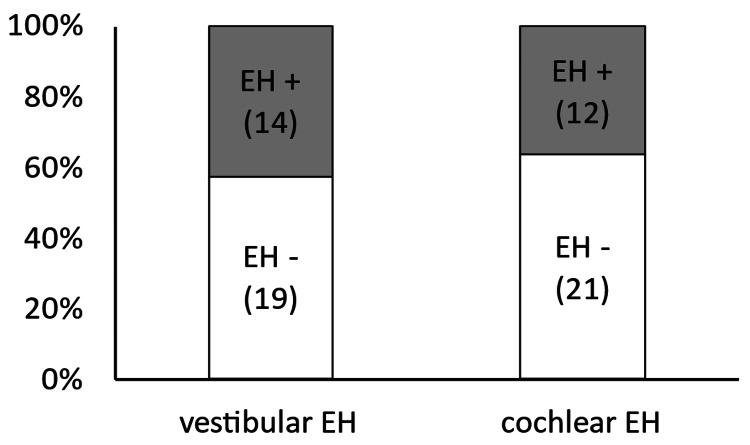
Consistency in the detection of endolymphatic hydrops with the VEMP On the affected side in the VEMP test, the ratios of the presence of vestibular and cochlear hydrops were similar (p = 0.80). EH, endolymphatic hydrops; VEMP, vestibular-evoked myogenic potential.

On the affected side in the VEMP test, the ratios of the presence of vestibular (14/33, 42.4%) and cochlear (12/33, 36.4%) hydrops were similar (p = 0.80) (Figure 5).

**Figure 6:**
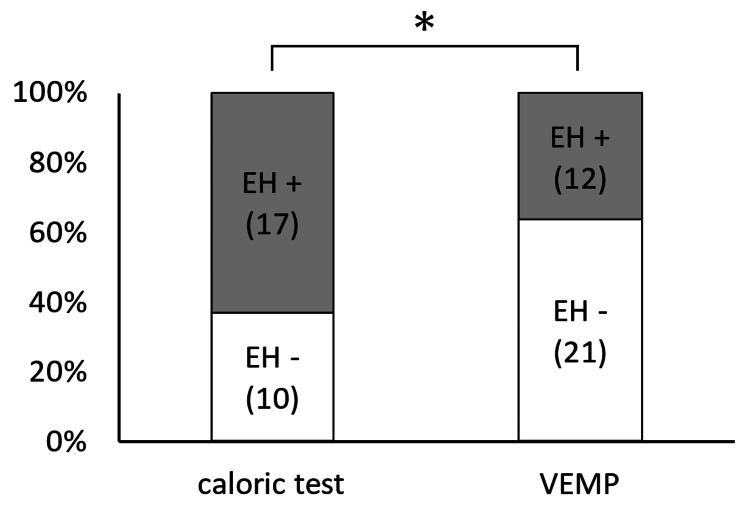
Consistency in the detection of cochlear endolymphatic hydrops with the caloric and VEMP test The consistency of the caloric test in detecting cochlear hydrops was higher than that of the VEMP test (p = 0.04). EH, endolymphatic hydrops; VEMP, vestibular-evoked myogenic potential; *, p < 0.05.

The consistency of the caloric test in detecting cochlear hydrops (17/27, 63.0%) was higher than that of the VEMP test (12/33, 36.4%) (p = 0.04) (Figure 6).

**Figure 7:**
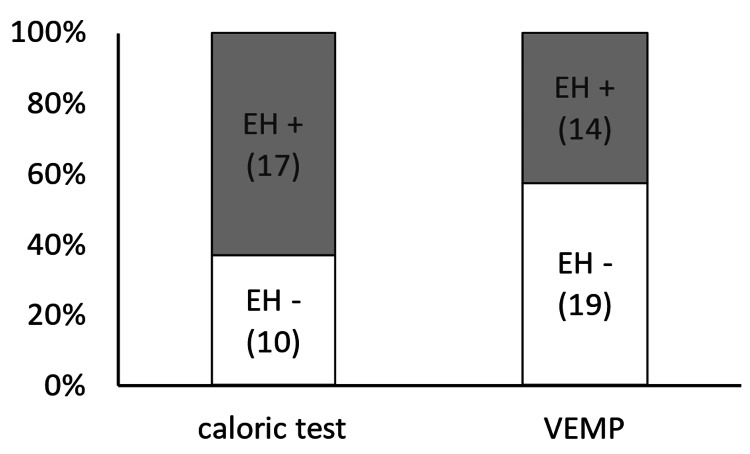
Consistency in the detection of vestibular endolymphatic hydrops with caloric and VEMP test The consistency of the caloric test in detecting vestibular hydrops tended to be higher (but not significantly) than that of the VEMP (p = 0.11). EH, endolymphatic hydrops; VEMP, vestibular-evoked myogenic potential.

Moreover, the consistency of the caloric test in detecting vestibular hydrops (17/27, 63.0%) tended to be higher (but not significantly) than that of the VEMP test (14/33, 42.4%) (p = 0.11) (Figure 7).

## Discussion

Our contrast-enhanced inner ear MRI revealed that 33 of the 49 patients (67.3%) had endolymphatic hydrops. Among them, 19 patients had bilateral endolymphatic hydrops, and 14 had unilateral hydrops. In previous reports, endolymphatic hydrops was observed on enhanced MRI in up to 90% of patients with possible and probable Meniere’s disease [7] and in all patients with definite Meniere’s disease [9-11]. The percentage of our patients with endolymphatic hydrops was smaller than that in previous reports, suggesting that a certain number of patients met the criteria for definite Meniere's disease but did not have endolymphatic hydrops. However, our diagnosis was based on the criteria defined by the AAO-HNS [2]. Patients who meet the diagnostic criteria for Meniere's disease but do not have endolymphatic hydrops and those with endolymphatic hydrops may have different diseases.

Duration of disease and MRI findings

The mean disease duration in patients with unilateral endolymphatic hydrops was longer than in patients with bilateral endolymphatic hydrops or without endolymphatic hydrops. Still, it has been reported that the incidence of bilaterally affected cases increases with age and the duration of Meniere’s disease [12]. The reason for this discrepancy is unknown; however, according to previous MRI analyses, endolymphatic hydrops is usually observed in the asymptomatic contralateral ear in 44-75% of patients [9,13]. Bilateral endolymphatic hydrops was observed in 19 of our patients; however, only four cases were confirmed as bilateral Meniere's disease. Therefore, the presence or absence of endolymphatic hydrops in the contralateral ear may not be critical.

Auditory tests and MRI findings

The mean hearing threshold and the sum of the three low frequencies of pure-tone audiometry were higher in patients with endolymphatic hydrops than in those without. According to previous reports, endolymphatic hydrops can progress during the disease course [14], and the extent of endolymphatic hydrops correlates with cochlear organ damage [4,5] and hearing impairment [12]. The audiometry results of our patients were consistent with those of previous studies. Positive glycerol test results were observed more frequently in patients with endolymphatic hydrops. This finding is consistent with glycerol administration improving hearing in patients with endolymphatic hydrops [8].

Vestibular tests and MRI findings

In the caloric and VEMP tests, the results in patients with endolymphatic hydrops and those in patients without hydrops did not differ. However, it has been reported that the severity of endolymphatic hydrops correlates with damage to vestibular organs [4,5]. According to a pathological study, endolymphatic hydrops is more frequently observed in the saccule and cochlea than in the utricle and semicircular canals [15]. In our patients, vestibular function was not as impaired by endolymphatic hydrops as cochlear function was, which differs from previous reports [4,5].

Correlation of the presence of endolymphatic hydrops with the clinically affected side

There is a high probability that endolymphatic hydrops is present in the cochlea/vestibule of at least the clinically affected side if limited to cases in which endolymphatic hydrops is identified. The percentage of our cases with endolymphatic hydrops on the clinically affected side (75%) was lower than that in the previous reports of patients with definite Meniere’s disease (100%) [9-11]. Irrespective of the presence or absence of endolymphatic hydrops on the unaffected side, there was a high probability of observing endolymphatic hydrops on the affected side.

Correlation of the presence of endolymphatic hydrops with the caloric and VEMP tests

It is assumed that the caloric and VEMP results are consistent with the presence of vestibular hydrops because the caloric and VEMP tests are vestibular function tests. However, for both tests, the concordance rates for the presence of cochlear and vestibular hydrops are comparable. There are some prior reports that examined the correlation between the findings of audio-vestibular function tests and the contrast-enhanced MRI findings in the inner ear [16-19]. However, no report mentioned that vestibular function tests predicted the presence of endolymphatic hydrops in the cochlea rather than the vestibule as in the present study.

For the presence of cochlear and vestibular hydrops, the concordance rate of the caloric results, i.e., percentage with both endolymphatic hydrops and abnormal vestibular function tests, tended to be higher than that of the VEMP results. In Meniere's disease, the caloric test (0.002-0.004 Hz) often shows a decreased response on the affected side; however, the video head impulse test (5-7 Hz) shows a normal response [20,21]. This means that type II hair cells, which are responsible for low frequencies, may be more likely to be damaged than type I hair cells, which are responsible for high frequencies. A guinea pig study of VEMPs with galvanic stimulation reported that VEMPs are heavily dependent on type I (and not type II) hair cell activity in the saccular macula [22]. In Meniere's disease, the VEMP test, which evaluates type I hair cell function, may be less impaired than the caloric test, which evaluates type II hair cell function. The concordance between VEMPs and the presence of endolymphatic hydrops might have been higher if glycerol-administered VEMP tests [23] or VEMP tuning property tests [24] were performed. However, these were not performed in this study because endolymphatic hydrops is more likely to occur in the saccule than in the semicircular canals [15].

This study has limitations. The number of subjects is not large, and the study needs to be developed with a larger number of subjects.

## Conclusions

The cochlea and vestibule on the clinically affected side were more likely to have endolymphatic hydrops revealed by contrast-enhanced 3-T inner ear MRI than on the unaffected side. The sum of the three low frequencies (125, 250, and 500 Hz) of the pure-tone audiometry was higher in patients with endolymphatic hydrops than in those without endolymphatic hydrops. The caloric test was more consistent in detecting endolymphatic hydrops, especially cochlear hydrops, than the VEMP test in patients with definite Meniere's disease. The results of this study may contribute to the future diagnosis of Meniere's disease and improve the understanding of endolymphatic hydrops.
